# Non-Linear and Sex-Specific Effect of Maternal Pre-Pregnancy BMI on Emotional and Behavioral Development of Preschool Children: A Population-Based Cohort Study

**DOI:** 10.3390/ijerph192013414

**Published:** 2022-10-17

**Authors:** Jingru Lu, Xuemei Hao, Linlin Zhu, Yufan Guo, Xiaoyan Wu, Jiahu Hao, Fangbiao Tao, Kun Huang

**Affiliations:** 1 Department of Maternal, Child and Adolescent Health, School of Public Health, Anhui Medical University, Hefei 230032, China; 2 Key Laboratory of Population Health Across Life Cycle, Ministry of Education of the People’s Republic of China, Anhui Medical University, Hefei 230032, China; 3 NHC Key Laboratory of Study on Abnormal Gametes and Reproductive Tract, Hefei 230032, China; 4 Anhui Provincial Key Laboratory of Population Health and Aristogenics, Hefei 230032, China; 5 Scientific Research Center in Preventive Medicine, School of Public Health, Anhui Medical University, Hefei 230032, China

**Keywords:** maternal pre-pregnancy weight, emotional and behavioral development, preschool children, sex difference

## Abstract

(1) Background: The aim was to examine the non-linear and sex-specific outcomes of maternal pre-pregnancy BMI on emotional and behavioral development of preschool children; (2) Methods: This study was based on the China-Anhui Birth Cohort (C-ABCS), including 3648 mother–child pairs. Maternal pre-pregnancy BMI was calculated from the maternal pre-pregnancy height and weight measured at the first antenatal checkup. Main caregivers completed the Strengths and Difficulties Questionnaire (SDQ) to assess children’s preschool emotional and behavioral development. A restricted cubic spline model was drawn using Stata version 15.1 to analyze the association between maternal pre-pregnancy BMI and preschoolers’ SDQ scores by sex; (3) Results: Among boys, maternal pre-pregnancy underweight was associated with the increased risk of conduct problems and pro-social behaviors, and pre-pregnancy overweight/obesity related with the increased risk of peer problems. Interestingly, when maternal pre-pregnancy BMI was between 18.50 kg/m^2^ and 18.67 kg/m^2^, boys had the increased risk of conduct problems. When pre-pregnancy BMI was between 18.50 kg/m^2^ and 19.57 kg/m^2^, boys had the increased risk of pro-social problems. No significant associations were observed; (4) Conclusions: A non-linear effect of maternal pre-pregnancy BMI on emotional and behavioral development has been found in preschool boys. In particular, pre-pregnancy normal weight may still affect boys’ emotional and behavioral development.

## 1. Introduction

Children’s emotional and behavioral problems (EBP) have attracted wide attention in recent years. Most EBPs occur in childhood or adolescence [1]. The 2016 National Survey of Children’s Health (NSCH) showed that among US children aged 3–17 years, 7.1% currently have anxiety problems, 3.2% currently have depression and 7.4% currently have behavioral/conduct problems [2]. It has been reported that the prevalence of children’s emotional and behavioral problems is 11.2–18.2% in China [3]. Without timely intervention, it will lead to long-term health issues such as anxiety and depression that persist into adulthood and affect social adjustment and life quality [4].

Maternal pre-pregnancy abnormal weight is common around the world. In Demographic and Health Surveys conducted in 24 countries, the prevalence of underweight in women before pregnancy was 10.9% [5]. It is estimated that by 2025, more than 21% of women will be obese in the world [6]. Being underweight or overweight before pregnancy would cause adverse pregnancy outcomes [7]. One study found that pre-pregnancy underweight significantly increased the risk of preterm birth, and pre-pregnancy obesity would lead to more than double the risk of pre-eclampsia and gestational diabetes [7]. Pre-pregnancy weight status can further affect offspring’s development, especially neurodevelopment [8]. Using Behavioral Problems Index (BPI) analysis, Julianna Deardorff et al. found that maternal pre-pregnancy underweight was associated with increased overall and externalizing problems in boys [8]. A meta-analysis showed that children whose mothers were overweight or obese before pregnancy were at increased risk for emotional and behavioral problems than children whose mothers were of normal weight before pregnancy [9].

Most of the existing studies have used body mass index (BMI) to reflect the maternal pre-pregnancy nutritional status. Although it can reflect the overall weight status, it cannot differentiate body composition, i.e., lean tissues and fat mass [10]. Adipose tissue is a vital component of the human body, but if in excess it can cause obesity, which represents a risk to an individual’s health [11]. Sometimes BMI is within normal range, but body fat (BF) has been excessive. Therefore, BMI within the normal range may also impact maternal and child health. A previous study found the association between maternal pre-pregnancy BMI and autism spectrum disorder (ASD) occurrence was non-linear and J-shaped, with extreme weight being associated with a higher risk of ASD compared to normal weight in the entire study population [12]. However, non-linear effects of pre-pregnancy BMI on general emotional and behavioral problems in offspring have not been reported. There is a significant sex difference in children’s emotional and behavioral development, boys are more likely to have behavioral problems and girls are more likely to have emotional problems[13,14]. However, sex differences have not been adequately considered when discussing the influence of pre-pregnancy weight status on offspring’s emotional and behavioral development in previous studies.

Based on a prospective, population-based birth cohort, we aim to examine the non-linear and sex-specific effect of maternal pre-pregnancy BMI on emotional and behavioral development of preschool children.

## 2. Materials and Methods

The current study was based on the China-Anhui Birth Cohort (C-ABCS). It is a population-based prospective cohort study focusing on the relationship between prenatal environmental exposures and child development. The cohort recruited 16,766 pregnant women from six major cities of Anhui Province in China between November 2008 and October 2010. Details of the cohort were reported elsewhere [15].

### 2.1. Participants

**Figure 1:**
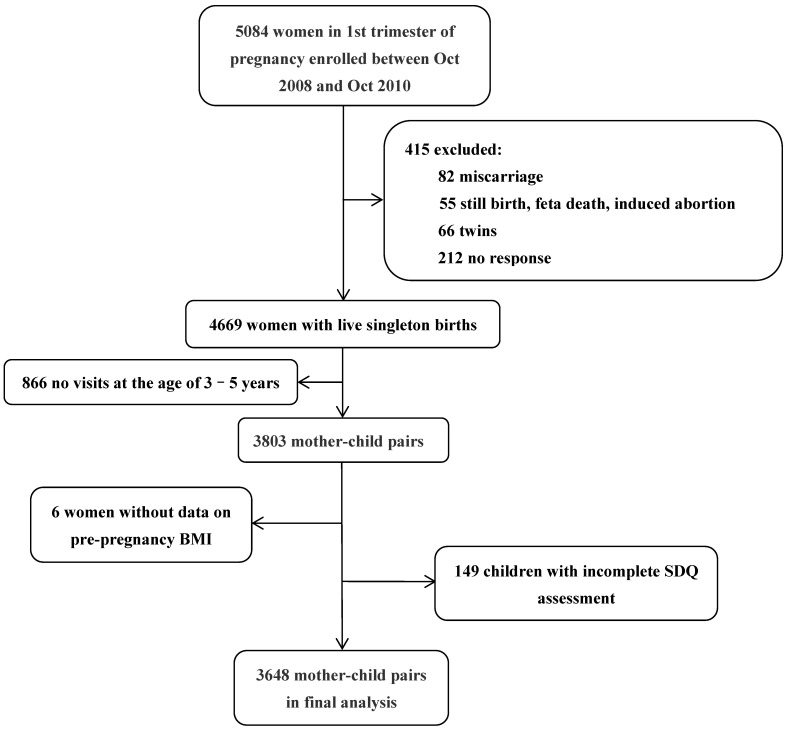
Flow chart of participant recruitment.

Pregnant women who came for their first antenatal visit to Ma’anshan Maternal and Child Health Care Hospital from November 2008 to October 2010 were recruited. The enrolling criteria included: (1) within 12 gestational weeks; (2) lived in Ma’anshan city for over six months; (3) being able to understand and complete questionnaires. A total of 5084 women were recruited. After excluding 137 adverse pregnancy outcomes (miscarriages, stillbirths, fetal death, induced abortion), 66 twins and 212 loss-to-follow up, a total of 4669 singleton live births were collected. Then, 866 no visits to children at the age of 3–5 years were excluded, 3803 mother–child pairs were included. Questionnaires are distributed to parents when they pick up their children and return to the nursery the following day. Six women with incomplete data on pre-pregnancy BMI and 149 children without emotional and behavioral assessments were further excluded. Finally, 3648 mother–child pairs with complete data were put into the final analysis. Among them, 1944 were boys, and 1704 were girls. The recruitment of participants is shown in Figure 1.

The study design was approved by the Biomedical Ethics Committee of Anhui Medical University (No. 2008020). All participants had signed informed consent.

### 2.2. Maternal Pre-Pregnancy BMI

Maternal height and weight were measured at the first antenatal visit. The weight was considered as pre-pregnancy weight. The mean gestational age at which BMI data were collected was (11.6 ± 2.5) weeks. Maternal pre-pregnancy BMI (kg/m^2^) was calculated by weight (kg)/height (m)^2^. According to the WHO classification criteria, maternal pre-pregnancy BMI categories were defined as underweight (BMI < 18.5 kg/m^2^), normal weight (18.5–24.99 kg/m^2^), overweight (25.0–29.99 kg/m^2^), and obese (≥30 kg/m^2^).

### 2.3. Assessment of Children’s Emotional and Behavioral Development

The Strengths and Difficulties Questionnaire (SDQ) completed by parents or other caregivers was used to assess children’s emotional and behavioral development. It contained 25 items which consisted of 5 sub-scales, including emotional symptoms, conductive problems, hyperactivity, peer relationship problems, and pro-social behaviors. Each item was scored as 0, 1, and 2 according to “not true”, “somewhat true”, and “completely true”. The former four sub-scales formed the Difficulties Questionnaire, which reflected negative emotions and behaviors. The sub-scale of pro-social behaviors was served as the Strengths Questionnaire, which referred to positive emotions and behaviors. The following cut-offs were used to identify abnormal emotional and behavioral development: emotional symptoms (≥5), conduct problems (≥4), hyperactivity (≥8), peer problems (≥6), total difficulties (≥17), and pro-social behaviors (≤4) [16].The previous study demonstrated that the internal consistency of the total difficulties score was good (Cronbach’s α, 0.78) [17]. Du et al. found that scores of SDQ closely related to the scores of Parent Symptoms Questionnaire (PSQ) in 1940 samples. They also assessed 47 Attention-deficit hyperactivity disorder (ADHD) children with SDQ and observed significant higher scores in these children than children without behavioral problems. It was verified that the convergent validity and discriminant validity of the Chinese-translated version were acceptable [16].

## 3. Covariates

**Figure 2:**
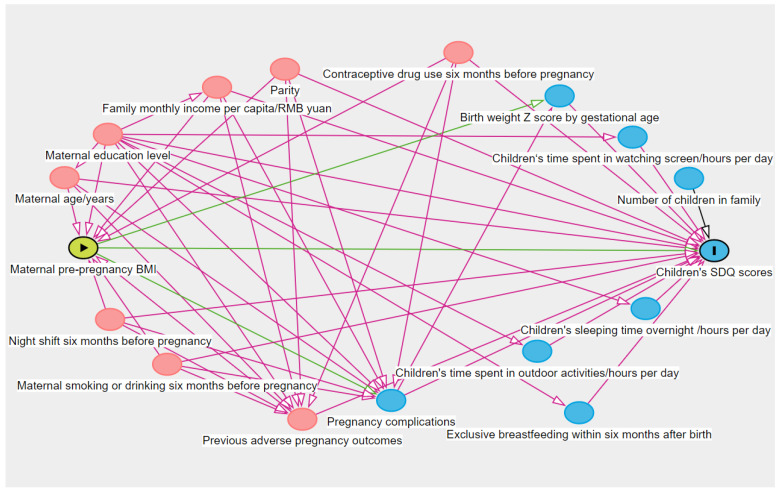
Directed Acyclic Graph of potential confounders.

The potential confounders were determined by using a literature review and Directed Acyclic Graph (DAG) [18] (Figure 2), including maternal age, maternal education level, family monthly income per capita, maternal night shift before pregnancy, women’s pre-pregnancy smoking and drinking, contraceptive drug use before pregnancy, previous adverse pregnancy outcomes and parity. The relevant data were obtained from the questionnaire survey during the first antenatal visit. Previous adverse pregnancy outcomes included spontaneous abortion, induced abortion, ectopic pregnancy, preterm birth, stillbirth, and having infants with birth defects. Women who experienced any of the above-mentioned conditions were defined as having previous adverse pregnancy outcomes. Smoking or drinking before pregnancy was defined as smoking or drinking by the pregnant woman six months before pregnancy. Smoking before pregnancy was reported by women in questionnaire, and the answers were set as “no smoking”, “less than one cigarette per day”, “1–5 cigarettes per day”, “6–10 cigarettes per day” and “more than half a pack per day”. Women who responded “no smoking” were defined as no pre-pregnancy smoking, otherwise as having pre-pregnancy smoking. Drinking before pregnancy was similarly defined, which was set as “never drinking”, “1–2 times a week,” and “3 times or more a week”.

Data on pregnancy complications, gestational age, birth weight, number of children in family, exclusive breastfeeding within six months after birth, children’s time spent in screening (cell phones, tablets, computers, etc.), children’s time spent in outdoor activities and children’s sleeping time overnight were collected for sensitivity analyses. This information was reported by children’s primary caregivers by filling in follow-up questionnaires when children aged 3 to 5. Exclusive breastfeeding within six months after birth was defined as infants who were exclusively breastfed and received no other foods or liquids besides [19]. Pregnancy complications covered hypertensive disorders and gestational diabetes. Women with either of the two conditions were regarded as having pregnancy complications. Children’s time spent in screening meant the average time spent by children on cell phones, tablets, and computers per day. Children’s time spent in outdoor activities meant the average time spent by children outdoor per day. Sleeping time overnight referred to the total time of children’s sleep at night per day, excluding nap time and time spent lying in bed ready for sleep.

### Statistical Analysis

EpiData 3.1 was used for data entry, and SPSS23.0 was used to analyze the data. Restricted cubic spline models were adopted using Stata 15.1, stratifying by children’s sex, to examine the association between maternal pre-pregnancy BMI and preschoolers’ emotional and behavioral development. The red lines in the figure indicated the odds ratios (ORs), and the solid black line indicated the 95% confidence intervals. The reference value of maternal pre-pregnancy BMI was set as 25 kg/m^2^, which is the dividing line between normal weight and overweight.

Four sensitivity analyses were performed. (1) High pre-pregnancy BMI may be related to maternal hypertensive disorders and gestational diabetes [20], and the two conditions would affect the offspring’s neurodevelopment [21,22]. Thus, pregnancy complications might be the potential mediator between the association of pre-pregnancy BMI and children’s emotional and behavioral problems and were further adjusted. (2) Studies have shown that children with low birth weight or born preterm are more likely to have behavioral and emotional problems [23,24]. Birth weight z-scores were thus adjusted in addition to the main analysis. The scores were calculated with the formula Z = (x − $\overset{\_}{x}$)/s, in which x was the children’s actual weight, $\overset{\_}{x}$ was the mean birth weight at each gestational week, and s was the standard deviation of birth weight at each gestational week. (3)WHO recommends for children’s exclusive breastfeeding within six months after birth [25], which is essential for children’s cognitive and social-emotional development. The variable was further included in the models. (4) Number of children in family [26], children’s time spent in screening [27], children’s time spent in outdoor activities [3], and children sleeping time overnight [28] were reported to be related to children’s emotional and behavioral development and were further adjusted as precision variables.

## 4. Results

### 4.1. Baseline Maternal and Children’s Characteristics

Baseline maternal and children’s characteristics are shown in Table 1. The mean maternal pre-pregnancy BMI was (20.2 ± 2.4) kg/m^2^ in mothers who had boys and (20.2 ± 2.4) kg/m^2^ in those who had girls. Totally 48% (1751/3648) of the pregnant women had adverse pregnancy outcomes, and 14.9% (531/3553) had pregnancy complications. Exclusive breastfeeding within six months accounted for 19.7% (705/3576) of children. Most children (92.1%) came from one-child families.

### 4.2. Prevalence of Children’s Abnormal SDQ Dimensions in Different Maternal Pre-Pregnancy BMI Categories

The prevalence of emotional symptoms, conduct problems, hyperactivity, peer problems, total difficulties and pro-social behavior was 4.9%, 8.1%, 9.0%, 3.8%, 7.9%, and 13.6% in boys and 7.8%, 6.9%, 6.1%, 1.8%, 7.3%, and 7.9% in girls, respectively.

The three nodes of maternal pre-pregnancy BMI, *P*_25_, *P*_50_, and *P*_75_, were used to describe the prevalence of children’s abnormal SDQ dimensions in different maternal pre-pregnancy BMI categories, which were divided into four groups: ≤*P*_25_, *P*_25_–*P*_50_, *P*_50_–*P*_75_, and >*P*_75_. The pre-pregnancy BMI of three nodes *P*_25_, *P*_50_ and *P*_75_ for mothers in boys were 18.64 kg/m^2^, 19.90 kg/m^2^, and 21.48 kg/m^2^, and the pre-pregnancy BMI of three nodes *P*_25_, *P*_50_ and *P*_75_ for mothers in girls were 18.55 kg/m^2^, 19.81 kg/m^2^, and 21.48 kg/m^2^ (Table 2).

### 4.3. Effect of Maternal Pre-Pregnancy BMI on Preschool Children’s Emotional and Behavioral Development

According to the four nodes of maternal pre-pregnancy BMI, *P*_5_, *P*_25_, *P*_75_, and *P*_95_, the restricted cubic spline plots of SDQ dimensions and maternal pre-pregnancy BMI for boys and girls were drawn, respectively. Maternal pre-pregnancy BMI of 25 kg/m^2^ was regarded as reference. Potential confounders were adjusted.

**Figure 3:**
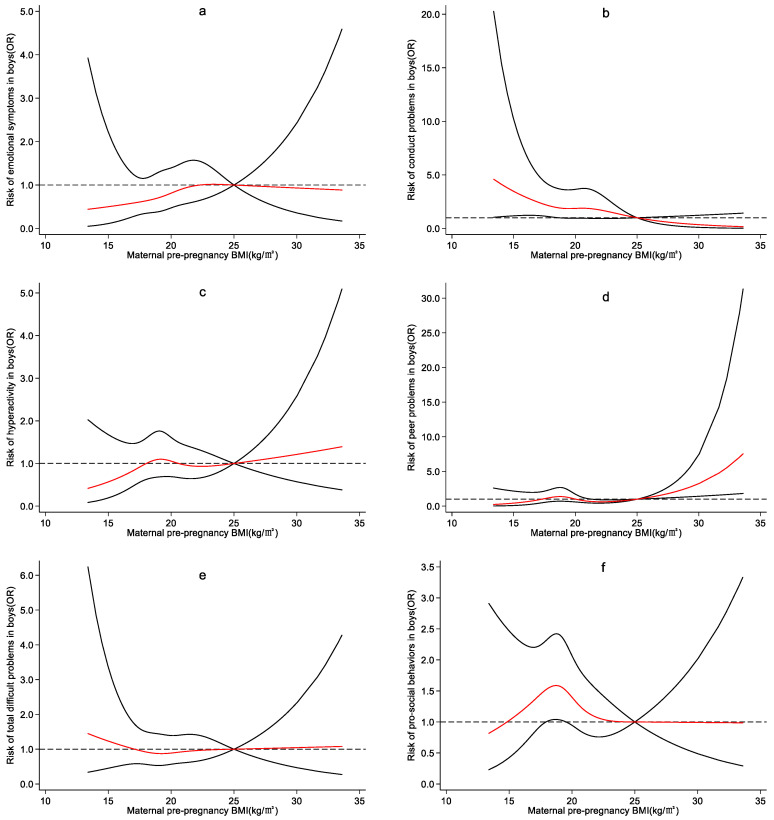
Nonlinear effect of maternal pre-pregnancy BMI on boys’ emotional and behavioral development. 1. Adjusting for maternal age, maternal education level, family monthly income per capita, maternal night shift before pregnancy, women’s pre-pregnancy smoking and drinking, contraceptive drug use before pregnancy, previous adverse pregnancy outcomes and parity. 2. The red lines in the figure indicated the odds ratios (ORs), and the solid black line indicated the 95% confidence intervals. 3. The reference value of maternal pre-pregnancy BMI was set as 25 kg/m^2^. 4. The subfigures (**a**–**f**) represent emotional symptoms, conductive problems, hyperactivity, peer problems, total difficult problems, and pro-social behaviors, respectively.

In boys, the risk of conduct problems increased when maternal pre-pregnancy BMI was between 13.38 kg/m^2^ and 18.67 kg/m^2^, OR (95%CI) being 4.60 (1.04–20.27) and 1.92(1.00–3.70), respectively (Figure 3b). When maternal pre-pregnancy BMI was between 25.04 kg/m^2^ and 33.59 kg/m^2^, the risk of peer problems increased, and OR (95%CI) was 1.01(1.00–1.02) and 7.56 (1.82–31.33), respectively (Figure 3d). Maternal pre-pregnancy BMI of 17.90–19.57 kg/m^2^ was associated with the risk of pro-social behaviors, and OR (95%CI) was 1.52(1.00–2.31) and 1.50 (1.00–2.25), respectively (Figure 3f).

**Figure 4:**
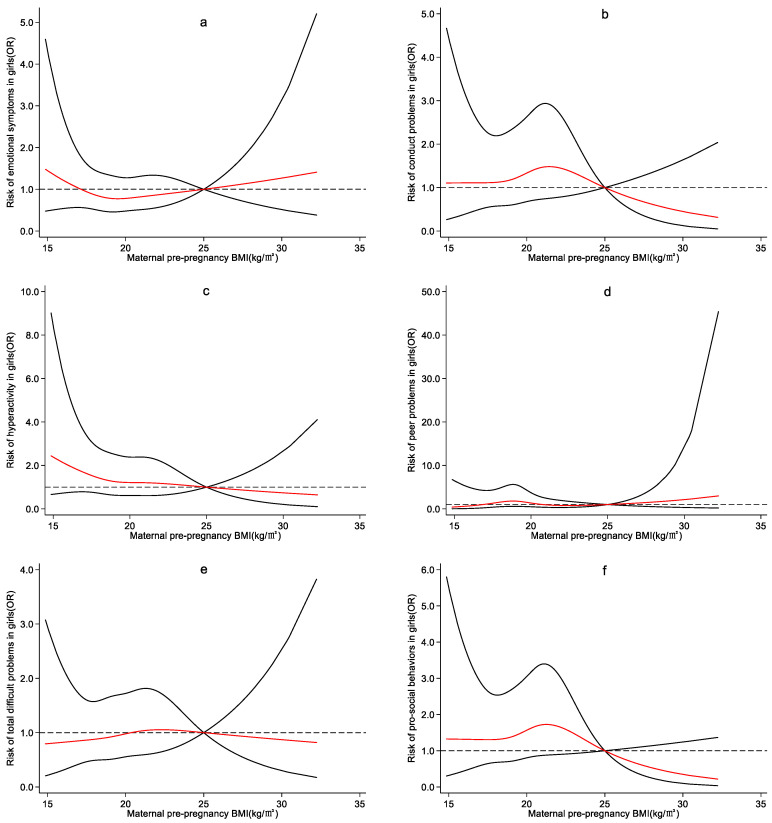
Nonlinear effect of maternal pre-pregnancy BMI on girls’ emotional and behavioral development. 1. adjusting for maternal age, maternal education level, family monthly income per capita, maternal night shift before pregnancy, women’s pre-pregnancy smoking and drinking, contraceptive drug use before pregnancy, previous adverse pregnancy outcomes and parity. 2. The red lines in the figure indicated the odds ratios (ORs), and the solid black line indicated the 95% confidence intervals. 3. The reference value of maternal pre-pregnancy BMI was set as 25 kg/m^2^. 4. The subfigures (**a**–**f**) represent emotional symptoms, conductive problems, hyperactivity, peer problems, total difficult problems, and pro-social behaviors, respectively.

No significant effect of maternal pre-pregnancy BMI on each dimension of SDQ in girls was observed (Figure 4).

**Figure A1:**
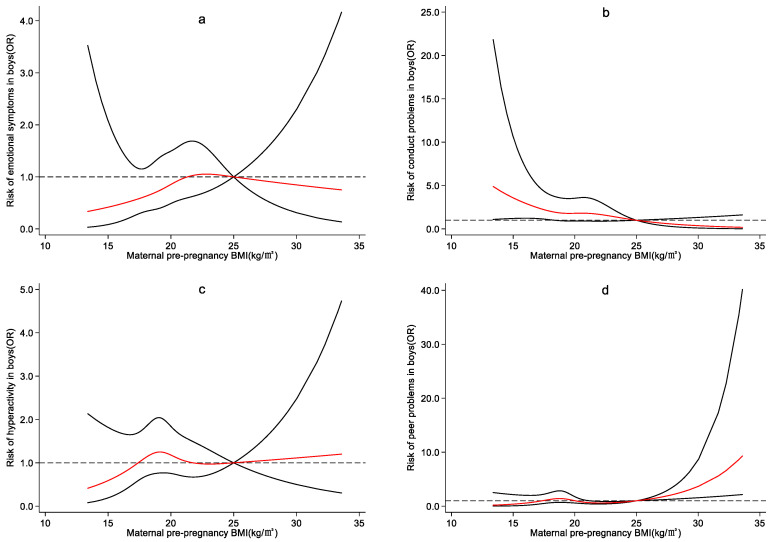

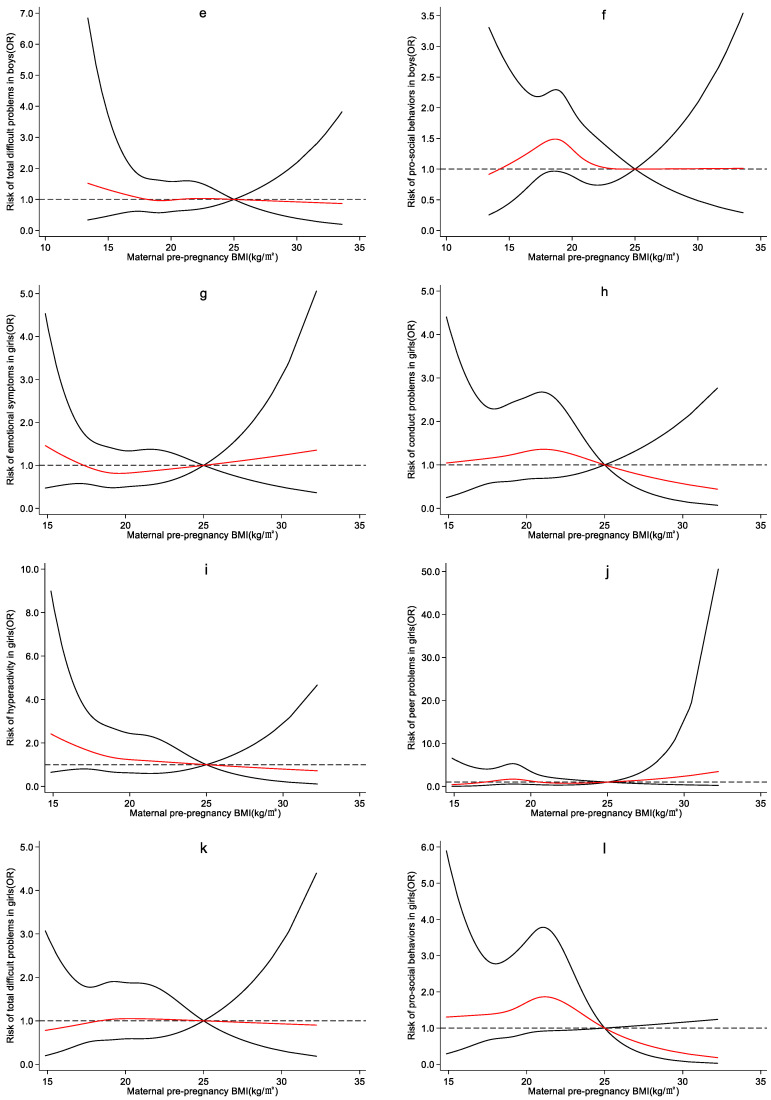
Sensitivity analysis 1—nonlinear effect of maternal pre-pregnancy BMI on children’ emotional and behavioral development. 1. adjusting for maternal age, maternal education level, family monthly income per capita, maternal night shift before pregnancy, women’s pre-pregnancy smoking and drinking, contraceptive drug use before pregnancy, previous adverse pregnancy outcomes, parity and pregnancy complications. 2. The red lines in the figure indicated the odds ratios (ORs), and the solid black line indicated the 95% confidence intervals. 3. The reference value of maternal pre-pregnancy BMI was set as 25 kg/m^2^. 4. The subfigures (**a**–**l**) represent emotional symptoms, conductive problems, hyperactivity, peer problems, total difficult problems, and pro-social behaviors, respectively.

**Figure A2:**
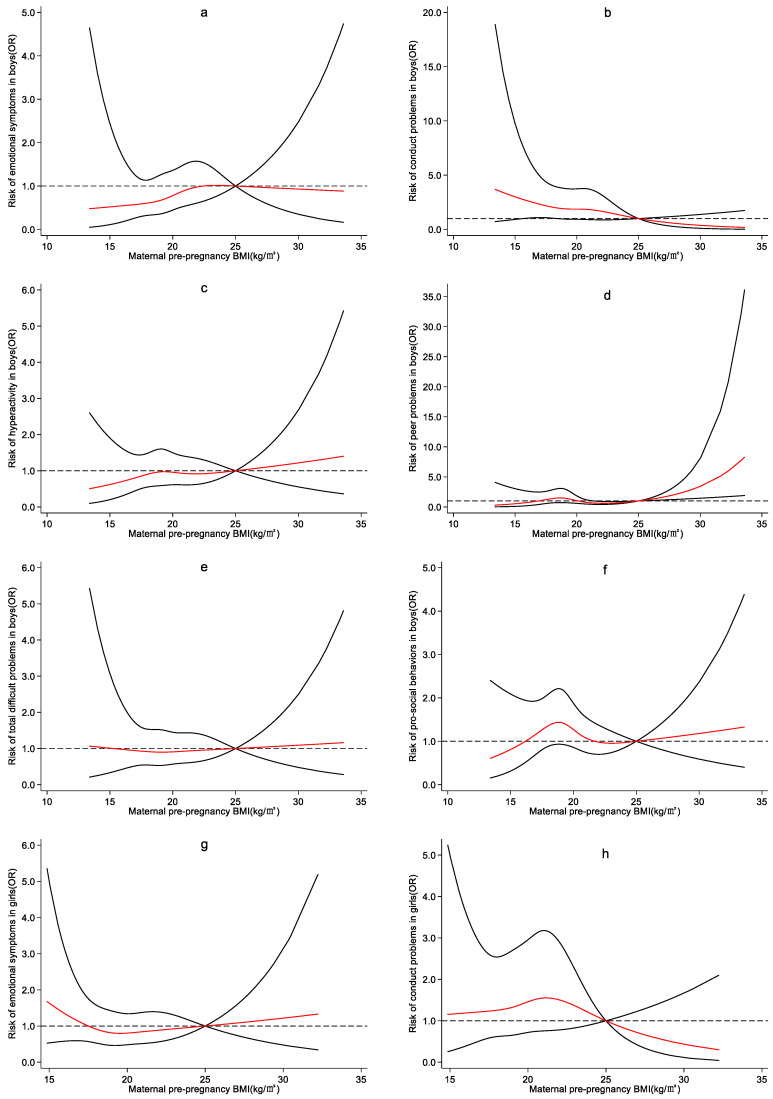

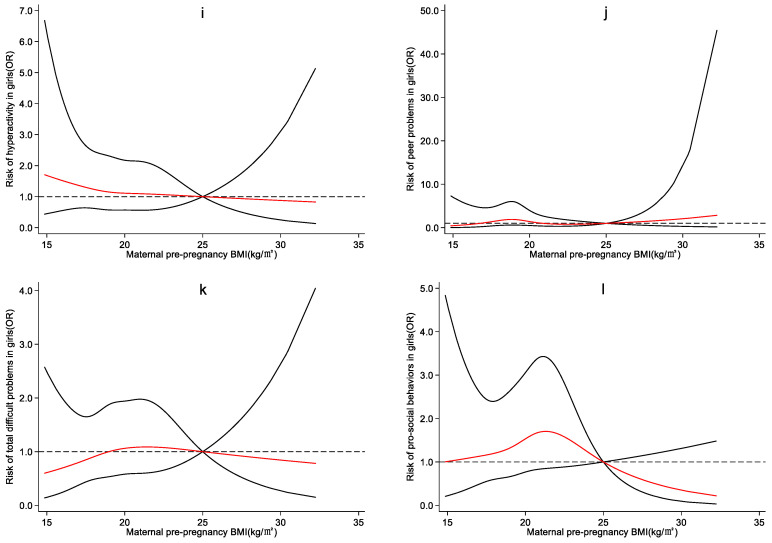
Sensitivity analysis 2—nonlinear effect of maternal pre-pregnancy BMI on children’ emotional and behavioral development. 1. adjusting for maternal age, maternal education level, family monthly income per capita, maternal night shift before pregnancy, women’s pre-pregnancy smoking and drinking, contraceptive drug use before pregnancy, previous adverse pregnancy outcomes, parity and birth weight Z scores by gestational age. 2. The red lines in the figure indicated the odds ratios (ORs), and the solid black line indicated the 95% confidence intervals. 3. The reference value of maternal pre-pregnancy BMI was set as 25 kg/m^2^. 4. The subfigures (**a**–**l**) represent emotional symptoms, conductive problems, hyperactivity, peer problems, total difficult problems, and pro-social behaviors, respectively.

**Figure A3:**
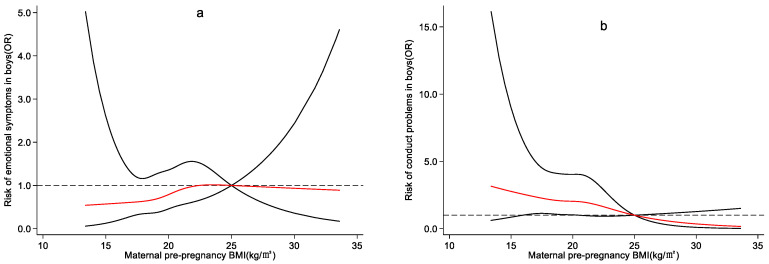

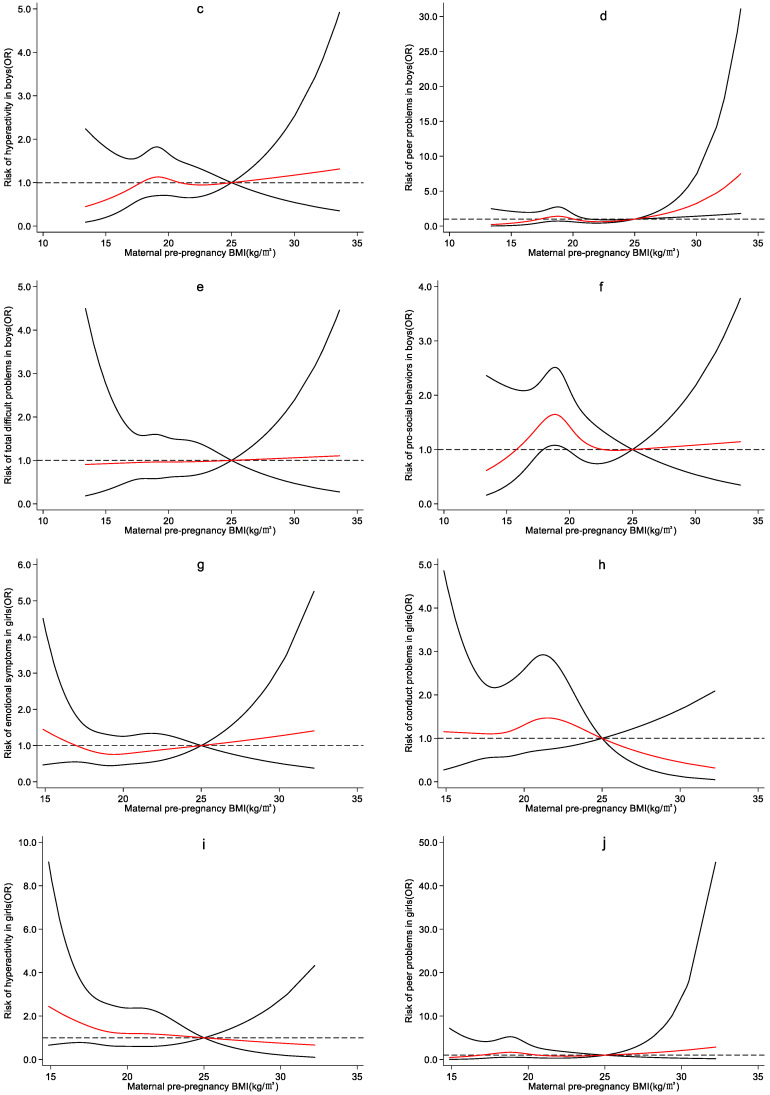

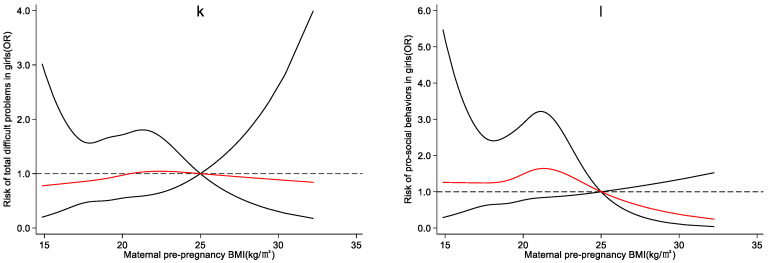
Sensitivity analysis 3—nonlinear effect of maternal pre-pregnancy BMI on children’ emotional and behavioral development. 1. adjusting for maternal age, maternal education level, family monthly income per capita, maternal night shift before pregnancy, women’s pre-pregnancy smoking and drinking, contraceptive drug use before pregnancy, previous adverse pregnancy outcomes, parity and exclusive breastfeeding within six months after birth. 2. The red lines in the figure indicated the odds ratios (ORs), and the solid black line indicated the 95% confidence intervals. 3. The reference value of maternal pre-pregnancy BMI was set as 25 kg/m^2^. 4. The subfigures (**a**–**l**) represent emotional symptoms, conductive problems, hyperactivity, peer problems, total difficult problems, and pro-social behaviors, respectively.

**Figure A4:**
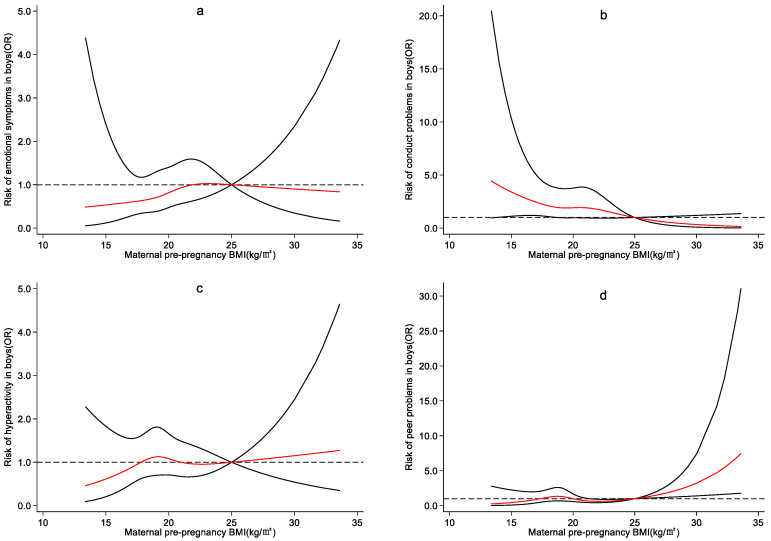

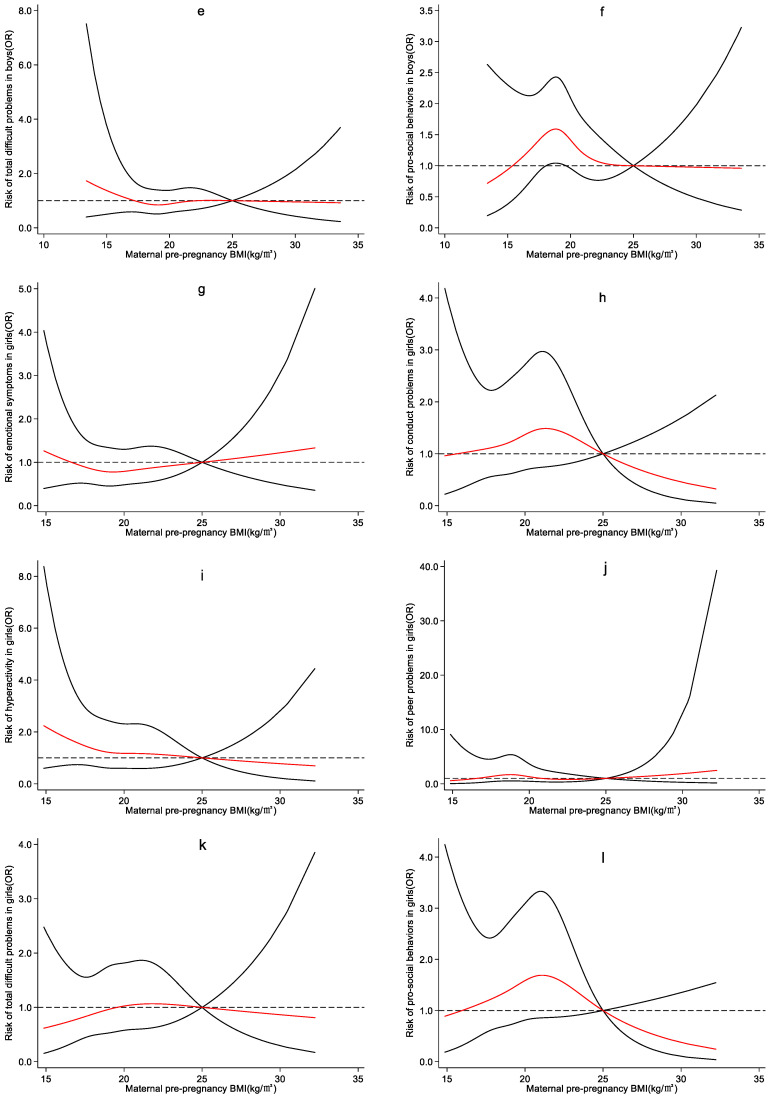
Sensitivity analysis 4—nonlinear effect of maternal pre-pregnancy BMI on children’ emotional and behavioral development. 1. adjusting for maternal age, maternal education level, family monthly income per capita, maternal night shift before pregnancy, women’s pre-pregnancy smoking and drinking, contraceptive drug use before pregnancy, previous adverse pregnancy outcomes, parity, the number of children in family, children’s time spent in watching screen, children’s time spent in outdoor activities and children sleeping time overnight. 2. The red lines in the figure indicated the odds ratios (ORs), and the solid black line indicated the 95% confidence intervals. 3. The reference value of maternal pre-pregnancy BMI was set as 25 kg/m^2^. 4. The subfigures (**a**–**l**) represent emotional symptoms, conductive problems, hyperactivity, peer problems, total difficult problems, and pro-social behaviors, respectively.

Sensitivity analyses did not fundamentally change the main findings (Figure A1, Figure A2, Figure A3 and Figure A4).

## 5. Discussion

In the current study, we found a non-linear effect of maternal pre-pregnancy BMI on boys’ emotional and behavioral development. Maternal pre-pregnancy underweight increased the risk of conduct problems and pro-social behaviors, and pre-pregnancy overweight/obesity increased the risk of peer problems. Interestingly, we found that although the maternal pre-pregnancy BMI was within the normal range, boys were still at increased risk of emotional and behavioral development problems. When BMI was between 18.5 kg/m^2^ and 18.67 kg/m^2^, boys were at increased risk of conduct problems. When BMI was between 18.5 kg/m^2^ and 19.57 kg/m^2^, boys were at increased risk of pro-social problems. In our study, we considered maternal weight at the first antenatal visit as the pre-pregnancy body weight. Women may experience minimal weight gain following conception to the time of their first antenatal visit, as pregnancy-related physiological conditions, such as nausea, vomiting might have acted to minimize weight gain [29]. So, it is suggested that there is acceptable concordance between pre-pregnancy BMI and BMI measured at the first antenatal visit [29].

Previous studies have shown that children born of mothers who are obese before pregnancy have an increased risk of behavioral problems compared to mothers with normal weight before pregnancy [30]. MENTING MD et al. found that maternal pre-pregnancy overweight/obesity was associated with an increased risk of children’s total behavioral problems and hyperactivity/inattention problems but not significantly associated with emotional, conduct and peer relationship problems in children [31], which was different from our findings.

The mechanism underlying the association between maternal pre-pregnancy obesity and offspring neurodevelopment is not clear. Maternal obesity may lead to elevated maternal IL-6 serum levels and IL-6 protein levels in adipose tissue [32]. Further studies have shown that elevated maternal serum IL-6 levels are associated with altered amygdala and brain connectivity in the offspring, which is common in children with neurodevelopmental disorders (NDDs) [33,34]. IL-6 and members of TGF and TNF families regulate many developmental processes from initial central nervous system (CNS) formation to synaptogenesis [35]. Dysregulation of these factors through maternal obesity may contribute to increased offspring’s susceptibility to neurodevelopmental disorders [36]. Animal experiments found increased circulating IL-6 and reduced innervation of the hypothalamic paraventricular nucleus in newborn mice of obese dams compared with those of dams with normal weight [37]. Increased maternal TNF-α expression due to maternal obesity may also affect offspring’s neurite growth [36]. Maternal metabolic disturbances due to obesity might affect microglial metabolic plasticity in the offspring, which would further impair neurodevelopment [36].

We also found that maternal pre-pregnancy underweight was associated with an increased risk of conduct problems and pro-social behaviors in boys. Currently, there are limited studies on the effects of maternal pre-pregnancy underweight on children’s emotional and behavioral development. In a developed society, being underweight is much less common than being overweight or obese. Previous studies have found that maternal pre-pregnancy underweight was associated with higher overall and externalizing problems in boys [8], as supported our findings. Evidence suggests that weight loss in women near conception may increase adverse birth outcomes [38]. Epidemiological studies have shown that pregnant women with low pre-pregnancy weight are at increased risk of preterm birth [39], which has been confirmed to be related with offspring’s long-term neurodevelopmental defects [40,41]. In the current study, maternal pre-pregnancy BMI was also significantly associated with boy’s preterm birth (*p* < 0.001). We hypothesize that maternal pre-pregnancy underweight may lead to preterm birth, which increases the risk of emotional and behavioral problems in preschool boys.

We also found that although the maternal pre-pregnancy BMI was within the normal range, boys were still at increased risk of emotional and behavioral development problems. It might be due to women’s excessive body fat and protein within normal BMI range. Of course, it is much suspected. In the future, BMI and body fat percentage (BF%) need to be integrated to evaluate maternal pre-pregnancy weight status.

A significant sex-specific effect of maternal pre-pregnancy BMI was observed on children’s emotional and behavioral development, primarily in boys. The underlying mechanisms are unclear. Animal and human studies have also confirmed that male fetuses may be more vulnerable to environmental exposures in utero [8]. Telomeres are variable numbers of complex nucleotide sequences that prevent chromosome degeneration and regulate cellular and tissue function [42]. Neonatal telomere length (TL) is a potential biomarker of prenatal exposure affecting offspring disease risk [43]. Pre-pregnancy BMI increased by 1 unit (1 kg/m^2^), the cord blood telomere length was shortened by 0.50%, and the placenta telomere length was shortened by 0.66% [44]. Short TL has been reported to be associated with depression, inattention, autism, and defiant behavior in later childhood and adolescence [45,46,47,48]. Elevated maternal pre-pregnancy BMI may be associated with short relative telomere length (rTL) in cord blood of male infants but not female newborns [43]. GRAF A E et.al constructed a model of pre-pregnancy obesity in dams through high fat diet (HFD). The study has found that decreases in myelination in the medial cortex were observed in male but not in female. The possible mechanism is that maternal pre-pregnancy obesity may induce neuroinflammatory responses in utero. Hepcidin signaling is reported to be increased in the setting of inflammation, which may cause disruption of iron regulation in male offspring’s brain and the subsequent neurobehavioral deficits [49].

### Strengths and Limitations

This study has several strengths. Firstly, we found a non-linear relationship between maternal pre-pregnancy weight and children’s emotional and behavioral development. Particularly, this study is the first to report that maternal pre-pregnancy BMI within the normal range increased the risk of boys’ emotional and behavioral problems. Meanwhile, this is a birth cohort study with a large sample and a high response rate (81.3%). Based on the prospective birth cohort, data on exposure, outcome and confounding factors could be accurately collected, and the recall bias could be effectively avoided. Thirdly, we fully considered the precision variables that would affect children’s emotional and behavioral development, which can further improve the accuracy of the findings.

This study also has serval limitations. Firstly, gestational weight gain (GWG) is closely related to pre-pregnancy BMI. Women with higher pre-pregnancy BMI may be likely to gain more weight during pregnancy than indicators issued by Institute of Medicine (IOM) guidelines [50]. We lack data on GWG, which is also associated with children’s neurodevelopment [51]. Meanwhile, the C-ABCS was the first cohort we have established in Anhui province, we did not collect maternal waist circumference and other measures of body fat. As we mentioned before, body fat is a better indicator of the body composition that can produce harmful effects than BMI. From May 2013 to September 2014, we have set up a second-round birth cohort. After fully considering that, maternal waist circumference by anthropometry and body fat by using a body composition analyzer was added in M-ABC. In the second, since maternal pre-pregnancy BMI data were collected between November 2008 and October 2010, there is a potential cohort effect. A Chinese study found that the rate of underweight decreased and the rate of overweight and obesity increased significantly among women of childbearing age between 2013 and 2019 in China [52]. Findings from the current study need to be interpreted prudently in the context of different maternal pre-pregnancy nutritional status. Thirdly, residual confounding cannot be fully ruled out. For instance, family structures [53] and the quality of pre-pregnancy diet [54] would influence children’s emotional and behavioral development. Finally, Finally, the use of informant-reported measures of health behaviors self-reported smoking history may be subject to recall bias [55], and direct measurement (such as nicotine) would be more realistic and objective. Similarly, informant-reported data might also influence SDQ findings. Children’s emotion and behavior have been reported by main caregivers in this study. Different caregivers may have different levels of concern about children’s emotional and behavioral problems. For example, compare with teachers, parents may be more inclined to report more emotional and behavioral problems [56]. Future studies should introduce emotional and behavioral assessment performed by teachers and child health professionals to decrease the potential effect.

## 6. Conclusions

A non-linear effect of maternal pre-pregnancy BMI on emotional and behavioral development has been found in preschool boys. In particular, pre-pregnancy normal weight may still affect boys’ emotional and behavioral development. This may be an effect of maternal body fat, and future studies are encouraged to combine BMI with body fat to investigate the effect on children’s brain development. It is better for women to have normal body weight and appropriate body fat mass. The sex difference in the effect of maternal pre-pregnancy BMI on children’s emotional and behavioral problems needs to be further verified by animal experiments.

## Figures and Tables

**Table 1:** Basic characteristics of participants.

Maternal and Children’s Characteristics	Boys (n = 1944)	Girls (n = 1704)	Statistical Value	***p*** **-Values**
** Mater ** **nal characteristic**				
Maternal age/years (M ± SD)	26.9 ± 3.4	26.8 ± 3.5	1.147	0.273
Maternal education level (n/%)				
Junior high school and below	459/23.6	408/23.9	1.128	0.770
High school or technical secondary school	561/28.9	465/27.3		
Junior college	507/26.1	455/26.7		
Bachelor degree and above	417/21.4	376/22.1		
Family monthly income per capita/RMB yuan (n/%) ^a^			4.759	0.093
<2000	1102/56.7	1022/60.0		
2000–4000	703/36.2	558/32.8		
>4000	139/7.1	123/7.2		
Parity (n/%)			2.077	0.150
Primiparity	1837/94.50	1628/95.5		
Multiparity	107/5.5	76/4.5		
Previous adverse pregnancy outcomes (n/%)			0.524	0.469
Did not have	1000/51.4	897/52.6		
Had	944/48.6	807/47.4		
Pregnancy complications (n/%) ^a^			0.138	0.710
Did not have	1607/84.8	1415/85.3		
Had	287/15.2	244/14.7		
Night shift six months before pregnancy (n/%)			1.107	0.293
Yes	325/16.7	263/15.4		
No	1619/83.3	1441/84.6		
Contraceptive drug use six months before pregnancy (n/%) ^a^			0.245	0.621
Yes	195/10.1	163/9.6		
No	1741/89.9	1538/90.4		
Maternal smoking six months before pregnancy (n/%)			0.701	0.403
Yes	80/4.1	61/3.6		
No	1864/95.9	1643/96.4		
Maternal drinking six months before pregnancy (n/%)			0.013	0.908
Yes	75/3.9	67/3.9		
No	1869/96.1	1637/96.1		
** Chil ** **dren’s characteristics**				
Gestational age/week (M±SD) ^a^	39.0 ± 1.3	39.1 ± 1.3	3.760	0.020
Birth weight/kg (M±SD) ^a^	3.5 ± 0.5	3.3 ± 0.4	2.411	<0.001
Exclusive breastfeeding within six months after birth (n/%) ^a^			0.081	0.777
Yes	372/19.5	333/19.9		
No	1532/80.5	1339/80.1		
Number of children in family (n/%)			4.991	0.025
One child	1791/92.1	1534/90.0		
More than one child	153/7.9	170/10.0		
Time spent in watching screen/hours per day (M ± SD)	0.7 ± 0.4	0.6 ± 0.4	3.313	<0.001
Time spent in outdoor activities/hours per day (M ± SD)	1.8 ± 1.0	1.6 ± 1.0	0.032	<0.001
Sleeping time overnight/hours per day ^a^ (M ± SD)	8.9 ± 0.8	9.0 ± 0.8	2.953	0.142

Abbreviations: M = mean; SD = standard deviation; “^a^” means missing value.

**Table 2:** Prevalence of children’s abnormal SDQ dimensions in different maternal pre-pregnancy BMI categories [n (%)].

Sex	Groups of Maternal Pre-Pregnancy BMI	Emotional Symptoms	Conduct Problems	Hyperactivity	Peer Problems	Total Difficult Problems	Pro-Social Behaviors
**Boys** **(n = 1944)**	≤18.64	20 (4.1)	47 (9.7)	44 (9.1)	21 (4.3)	42 (8.6)	71 (14.6)
	18.65–19.90	18 (3.7)	41 (8.4)	49 (10.0)	19 (3.9)	37 (7.6)	72 (14.8)
	19.91–21.48	33 (6.6)	40 (8.0)	35 (7.0)	17 (3.4)	35 (7.0)	72 (14.3)
	≥21.49	25 (5.4)	29 (6.2)	46 (9.9)	16 (3.4)	39 (8.4)	49 (10.5)
**Girls** **(n = 1704)**	≤18.55	38 (8.8)	30 (7.0)	33 (7.7)	8 (1.9)	30 (7.0)	28 (6.5)
	18.56–19.81	29 (6.9)	28 (6.6)	23 (5.4)	10 (2.4)	26 (6.1)	33 (7.8)
	19.82–21.48	32 (7.2)	30 (6.8)	32 (7.2)	7 (1.6)	39 (8.8)	43 (9.7)
	≥21.49	34 (8.4)	29 (7.1)	16 (3.9)	5 (1.2)	29 (7.1)	30 (7.4)

## Data Availability

The data are not publicly available due to privacy restrictions. If required, the data that support the findings of this study are available on request from the corresponding author.
